# Streptococcus gordonii Type I Lipoteichoic Acid Contributes to Surface Protein Biogenesis

**DOI:** 10.1128/mSphere.00814-19

**Published:** 2019-12-04

**Authors:** Bruno P. Lima, Kelvin Kho, Brittany L. Nairn, Julia R. Davies, Gunnel Svensäter, Ruoqiong Chen, Amanda Steffes, Gerrit W. Vreeman, Timothy C. Meredith, Mark C. Herzberg

**Affiliations:** a Department of Diagnostic and Biological Sciences, School of Dentistry, University of Minnesota, Minneapolis, Minnesota, USA; b Department of Oral Biology, Faculty of Odontology, Malmo University, Malmo, Sweden; c Department of Biochemistry and Molecular Biology, The Pennsylvania State University, State College, Pennsylvania, USA; University of Iowa

**Keywords:** Gram-positive bacteria, LTA, *Streptococcus gordonii*, cell wall, lipoteichoic acid, surface proteins

## Abstract

Lipoteichoic acid (LTA) is an abundant polymer of the Gram-positive bacterial cell envelope and is essential for many species. Whereas the exact function of LTA has not been elucidated, loss of LTA in some species affects hydrophobicity, biofilm formation, and cell division. Using a viable LTA-deficient strain of the human oral commensal Streptococcus gordonii, we demonstrated that LTA plays an important role in surface protein presentation. Cell wall fractions derived from the wild-type and LTA-deficient strains of S. gordonii were analyzed using label-free mass spectroscopy. Comparisons showed that the abundances of many proteins differed, including (i) SspA, SspB, and S. gordonii 0707 (SGO_0707) (biofilm formation); (ii) FtsE (cell division); (iii) Pbp1a and Pbp2a (cell wall biosynthesis and remodeling); and (iv) DegP (envelope stress response). These changes in cell surface protein presentation appear to explain our observations of altered cell envelope homeostasis, biofilm formation, and adhesion to eukaryotic cells, without affecting binding and coaggregation with other bacterial species, and provide insight into the phenotypes revealed by the loss of LTA in other species of Gram-positive bacteria. We also characterized the chemical structure of the LTA expressed by S. gordonii. Similarly to Streptococcus suis, S. gordonii produced a complex type I LTA, decorated with multiple d-alanylations and glycosylations. Hence, the S. gordonii LTA appears to orchestrate expression and presentation of cell surface-associated proteins and functions.

**IMPORTANCE** Discovered over a half-century ago, lipoteichoic acid (LTA) is an abundant polymer found on the surface of Gram-positive bacteria. Although LTA is essential for the survival of many Gram-positive species, knowledge of how LTA contributes to bacterial physiology has remained elusive. Recently, LTA-deficient strains have been generated in some Gram-positive species, including the human oral commensal Streptococcus gordonii. The significance of our research is that we utilized an LTA-deficient strain of S. gordonii to address why LTA is physiologically important to Gram-positive bacteria. We demonstrate that in S. gordonii, LTA plays an important role in the presentation of many cell surface-associated proteins, contributing to cell envelope homeostasis, cell-to-cell interactions in biofilms, and adhesion to eukaryotic cells. These data may broadly reflect a physiological role of LTA in Gram-positive bacteria.

## Abstract (precis)

Discovered over a half-century ago, lipoteichoic acid (LTA) is an abundant polymer found on the surface of Gram-positive bacteria. Although LTA is essential for the survival of many Gram-positive species, knowledge of how LTA contributes to bacterial physiology has remained elusive. Recently, LTA-deficient strains have been generated in some Gram-positive species, including the human oral commensal Streptococcus gordonii. The significance of our research is that we utilized an LTA-deficient strain of S. gordonii to address why LTA is physiologically important to Gram-positive bacteria. We demonstrate that in S. gordonii, LTA plays an important role in the presentation of many cell surface-associated proteins, contributing to cell envelope homeostasis, cell-to-cell interactions in biofilms, and adhesion to eukaryotic cells. These data may broadly reflect a physiological role of LTA in Gram-positive bacteria.

## INTRODUCTION

Bacteria interact directly with their environment through surface structures that decorate the cell envelope. The cell envelope of most Gram-positive bacteria consists of the exterior, thick peptidoglycan layer of the cell wall and the interior cytoplasmic membrane. Essential to cell physiology, outward-reaching structures anchored to the cell envelope mediate surface attachment and interspecies interactions, protect against environmental stresses, and help maintain cellular homeostasis (1).

Among the surface polymers of the cell envelope are exterior-reaching glycolipid polymers, including teichoic acids (TAs), which contain phosphodiester-linked polyol repeat units (2). Discovered almost 60 years ago (3), TAs can be found attached to the cell wall (wall teichoic acid [WTA]) or to the cell membrane (lipoteichoic acid [LTA]). Based on their chemical composition, five types of LTAs (types I to V) have been characterized (reviewed in reference 4).

LTA polymers are abundant and comprise a significant percentage of the dry weight of the bacterial cell wall (5). Cells lacking LTA are often nonviable or display severe growth defects (6). LTA contributes to surface hydrophobicity (7, 8), Mg^2+^ ion scavenging (9), and cell division (10). LTA may also function as an adhesion-promoting molecule (adhesin), mediating interactions with other bacterial and/or eukaryotic cells (11, 12). Thus, LTA is presumed to play an important role in the overall biology of Gram-positive bacteria. The precise physiological function(s) of LTA in the bacterial cell, however, remains unknown.

Recently, a viable LTA-defective strain of the human oral commensal Streptococcus gordonii was generated and studied for its role in the production of nitric oxide by murine macrophages (13) and of interleukin-8 by human periodontal ligament cells (14). A commensal bacterium and pioneer colonizer in oral biofilms, including dental plaque (15), S. gordonii belongs to the viridans group of oral streptococci (16). In dental plaque, S. gordonii is considered beneficial, antagonizing its cariogenic relative, Streptococcus mutans (17). In the bloodstream, however, endogenous strains of S. gordonii have been associated with systemic infections, including infective endocarditis (18, 19).

To investigate how LTA impacts the physiology of S. gordonii cells, we constructed a viable LTA-deficient strain. Here, we report that LTA plays a significant role in surface protein biogenesis, affecting the presentation of several cell wall-associated proteins. These proteins are involved in surface attachment, cell division, and peptidoglycan synthesis, ultimately affecting biofilm formation and the ability of S. gordonii to bind oral keratinocytes but not coaggregation with other bacterial species.

## RESULTS

### Mutant confirmation.

**FIG 1:**
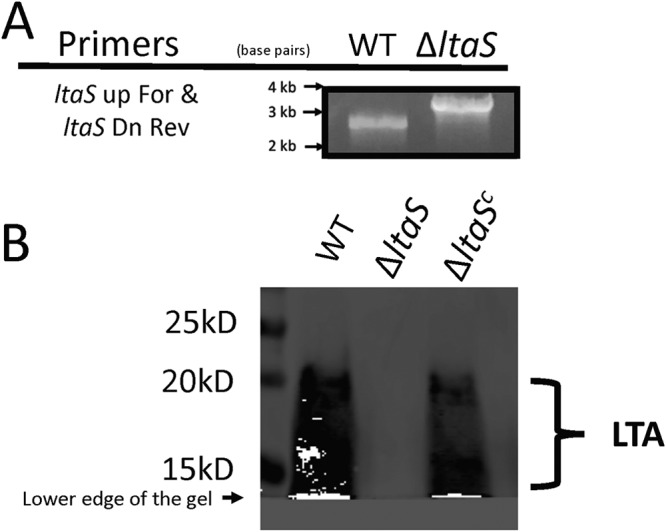
*ltaS* deletion leads to loss of LTA. (A) *ltaS* deletion was confirmed by PCR amplification of the wild-type (WT) and the LTA-deficient (Δ*ltaS*) genomic DNA with the primer pair *ltaS* up For and *ltaS* Dn Rev. (B) Mouse anti-LTA antibody was used to detect LTA presence on cell wall fractions from the WT strain, the Δ*ltaS* strain, and the complemented LTA deletion strain (Δ*ltaS^c^*) using Western blotting.

An LTA-deficient strain of S. gordonii DL1 was constructed by allelic replacement of an open reading frame (S. gordonii 1377 [*SGO_1377*]) which encodes the LTA synthase (LtaS) homolog in S. gordonii*. SGO_1377* replacement (Δ*ltaS*) was confirmed by PCR (Fig. 1A) and whole-genome sequence analysis (see Fig. S1 in the supplemental material). A monoclonal antibody against LTA was used to confirm the loss of LTA in the *SGO_1377* deletion background (Fig. 1B). LTA synthesis was restored when *SGO_1377* was reinserted into the chromosome at the *attB* site (Δ*ltaS^c^*) (Fig. 1B). SGO_1377 is 39% identical and 57% similar to LtaS of Staphylococcus aureus Newman. Since it is required for LTA synthesis by S. gordonii, SGO_1377 is referred to here as LtaS.

10.1128/mSphere.00814-19.1 FIG S1 Whole-genome sequencing confirms *ltaS* deletion. The image shows the region of the S. gordonii genome at the *ltaS* locus in the WT or Δ*ltaS* strains. The Δ*ltaS* strain showed an absence of sequence alignment at the correct locus when aligned with the reference genome and WT strain. Download FIG S1, TIF file, 0.2 MB. Copyright © 2019 Lima et al. 2019 Lima et al. This content is distributed under the terms of the Creative Commons Attribution 4.0 International license.

### S. gordonii LTA structure.

**FIG 2:**
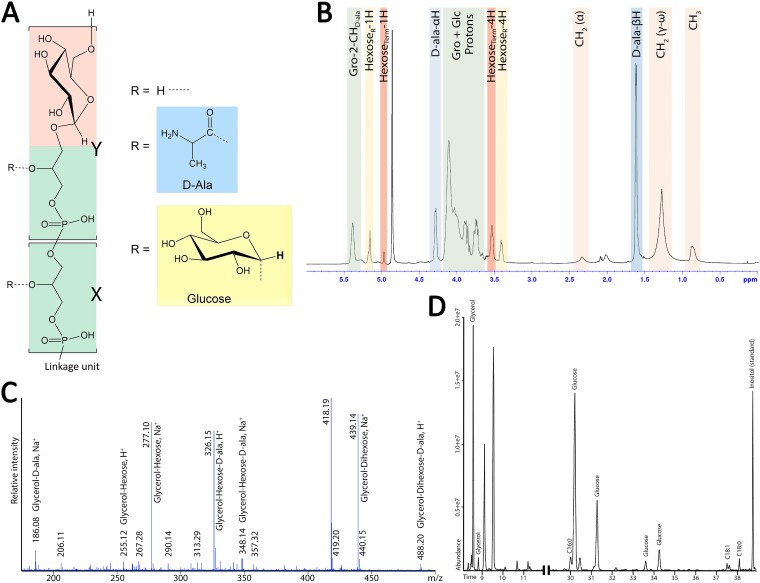
LTA structure. (A) Proposed structure of S. gordonii type I LTA with glycerol phosphate (X) and terminal glucose-glycerol phosphate (Y) repeat units. (B) NMR spectrum of purified LTA extracted from S. gordonii containing two distinct anomeric proton signals. (C) Purified S. gordonii LTA was also subjected to electrospray ionization-mass spectrometry after monomerization by hydrofluoric acid. Multiply modified glycerol signals are observed at *m/z* 326.15, 348.14, 439.14, and 488.20. (D) Carbohydrate composition analysis of monomerized LTA by GC/MS (carbohydrate composition) identified glucose as the sole monosaccharide residue in the S. gordonii LTA, comprising 43.2% (mol%) versus 56.8% of glycerol.

Five types of LTAs (types I to V) have been described based on the chemical architecture of their repeating units (e.g., polyglycerolphosphate [type I], complex glycosylglycerol-phosphate [types II and III], glycosyl-ribitolphosphate [type IV], and glycosyl-phosphate [type V]) (4). The S. gordonii LTA is consistent with the Streptococcus suis type I LTA decorated with d-alanines and glycosyls as analyzed by one-dimensional (1D) ^1^H nuclear magnetic resonance (NMR) (Fig. 2A). The signals at 5.16 ppm and 3.41 ppm are consistent with hexose anomeric H-1 and H-4 chemical shifts. The second set of signals at 4.97 ppm and 3.54 ppm suggests alternative glycosylation corresponding to either a different sugar moiety or a different position of the LTA backbone (Fig. 2B). To better resolve the structure, purified LTA monomers were subjected to electrospray ionization-mass spectrometry (ESI-MS). Consistent with the presence of hexose substituents, ion signals corresponding to glycerol-hexose were observed at *m/z* 255.12 (H^+^) and 277.10 (Na^+^). Abundant ion signals were also observed, corresponding to multiple glycerol modifications (e.g., *m/z* 326.15 [glycerol-hexose-d-ala, H^+^], 348.14 [glycerol-hexose-d-ala, Na^+^], and 439.14 [glycerol-dihexose, Na^+^]) (Fig. 2C). Moreover, data from glycosyl compositional analysis indicate that glucose is the sole, highly abundant LTA-associated monosaccharide residue, strongly suggesting that the glycerol repeat units contain at least two unique glycosidic linkages involving glucose (Fig. 2D). As is common with LTA lipid anchors, fatty acid heterogeneity was observed in the forms of C16:0, C18:1, and C18:0.

The ^1^H NMR spectrum, including the two distinct α-glucose anomeric proton signals at 4.97 ppm and 5.16 ppm, is consistent with the type I LTA structure recently characterized in S. suis serotype 2 strain 89-1591 (ST25) (20). The ST25 LTA is a complex type I LTA. Terminal glycerol-glucose repeating units cap a simple type I glycerol phosphate repeating unit. Both repeat units can still be modified by d-alanylation or glycosylation on the glycerol C2-OH position, hence, the presence of the signals of the multiply modified glycerols.

### Growth and morphological effects. (i) Growth.

**FIG 3:**
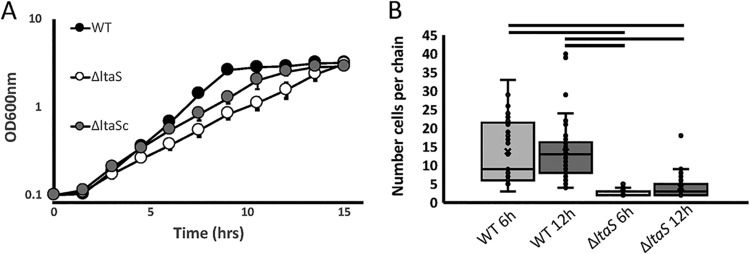
*ltaS* deletion affects growth and chain length. (A) Growth curve of S. gordonii strains (WT, Δ*ltaS*, and Δ*ltaS^c^*) as determined by optical density (λ = 600 nm). Data represent means of results from three independent biological replicates (± standard deviations [SD]). (B) Number of cells per chain of S. gordonii in samples from WT and Δ*ltaS* strains collected after 6 and 12 h of growth visualized by light microscopy. Each data point represents one chain. Bars on top of the graph show statistical significance (*P* < 0.05) as determined by analysis of variance (ANOVA).

The *ΔltaS* strain grew more slowly than the wild type (WT) on solid media. An additional day of incubation was needed before colonies were visualized on the antibiotic-selection plates. The Δ*ltaS* cells showed a longer doubling time (∼145 min) than the WT cells (∼85 min), whereas the two strains grew to similar densities at the stationary phase (15 h) in FMC liquid medium (Fig. 3A), suggesting that although loss of LTA affects cell division, the mutant and WT can grow to similar cell densities. Complementation of the Δ*ltaS* strain (strain *ΔltaS^c^*) partially rescued the growth defect (Fig. 3A).

**FIG 4:**
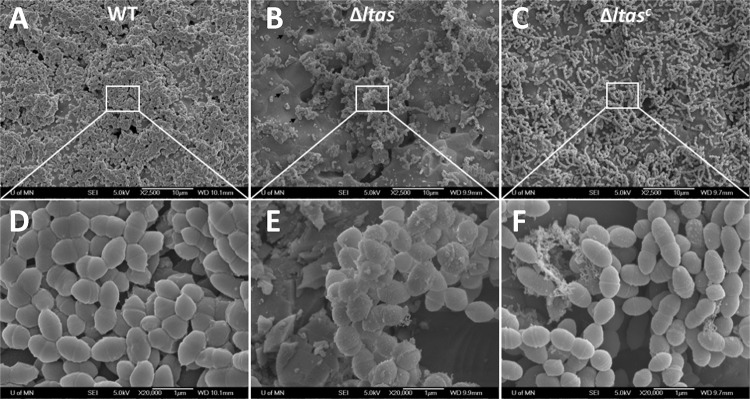
SEM of S. gordonii biofilms. S. gordonii strains (WT, Δ*ltaS*, and Δ*ltaS^c^*) were allowed to form biofilm on saliva-coated hydroxyapatite disks for 12 h. Biofilms were visualized at ×2,500 (A [WT strain], B [Δ*ltaS* strain], and C [Δ*ltaS^c^* strain]) and ×20,000 (D [WT strain], E [Δ*ltaS* strain], and F [Δ*ltaS^c^* strain]).

**(ii) Coccal chain size and cellular morphology.** At 6 h (mid-exponential phase) and 12 h (stationary phase) of growth, the Δ*ltaS* mutant grew in shorter chains than the WT (Fig. 3B). Since LTA is an abundant component of the Gram-positive cell wall, changes in morphology due to loss of LTA were visualized in biofilms using scanning electron microscopy (SEM). After growth on saliva-coated hydroxyapatite disks for 12 h, the WT strains formed a thick biofilm that covered most of the disk surface (Fig. 4A), while the Δ*ltaS* strain failed to cover the disk surface (Fig. 4B). Complementation of the Δ*ltaS* strain partially restored the biofilm phenotype (Fig. 4C).

At higher magnification (×20,000), the surface of the WT cells appeared smooth and uniform (Fig. 4D; see also Fig. S2A), whereas the cell surface of the Δ*ltaS* strain appeared irregular, and amorphous extracellular material was apparent (Fig. 4E; see also Fig. S2B). The cell surface of the complemented strain was more similar to the cell surface of the WT strain than to that of the Δ*ltaS* strain (Fig. 4F). The Δ*ltaS* cells appeared rounder than the WT cells (Fig. S2A and B), perhaps due to a weaker peptidoglycan layer. Consistent with a decrease in cell wall strength, the Δ*ltaS* mutant was significantly more sensitive to exposure to hypotonic shock than the WT (Fig. S3).

10.1128/mSphere.00814-19.2 FIG S2 S. gordonii strains (WT and Δ*ltaS*) were allowed to form biofilm on saliva-coated hydroxyapatite disks for 6 h. Biofilms were visualized at ×5,000. Download FIG S2, TIF file, 0.8 MB. Copyright © 2019 Lima et al. 2019 Lima et al. This content is distributed under the terms of the Creative Commons Attribution 4.0 International license.

10.1128/mSphere.00814-19.3 FIG S3 Equal numbers of WT and Δ*ltaS* S. gordonii cells collected during mid-exponential phase were exposed to sterile PBS or water for 30 min. Next, the cells were plated on TH agar (A) and the colonies were enumerated to determine the percentage of surviving cells compared to the initial inoculum (B). Bars represent means of results from six biological replicates ± SD. Download FIG S3, JPG file, 0.03 MB. Copyright © 2019 Lima et al. 2019 Lima et al. This content is distributed under the terms of the Creative Commons Attribution 4.0 International license.

### Functional effects. (i) Surface attachment and biofilm.

**FIG 5:**
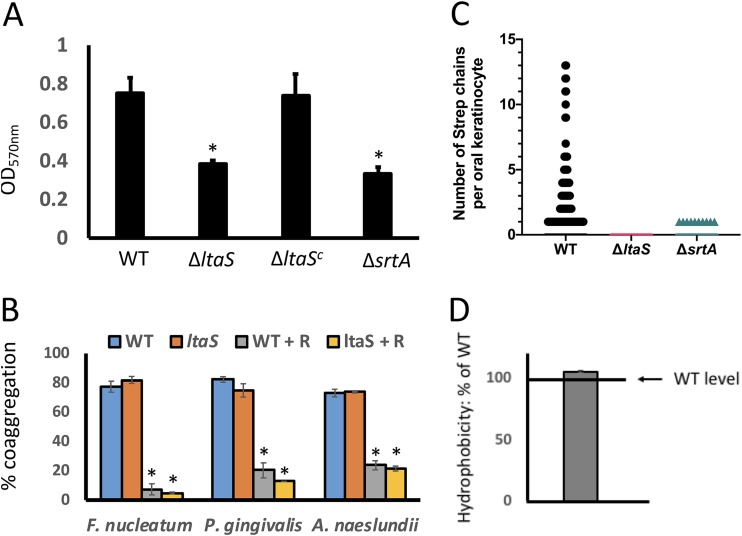
LTA is involved in biofilm formation and interaction with oral keratinocytes. (A) Quantification of biofilm biomass of S. gordonii WT, Δ*ltaS*, Δ*ltaS^c^*, and Δ*srtA* strains on saliva-coated polystyrene wells. Bars represent means of results from six biological replicates ± SD. *, *P* < 0.05. (B) Quantitative coaggregation of S. gordonii WT and Δ*ltaS* strains with F. nucleatum, P. gingivalis, and A. naeslundii with or without 50 mM l-arginine (R). Bars represent means of results from six biological replicates ± SD. *, *P* < 0.05. (C) Number of S. gordonii cell chains from the WT, Δ*ltaS*, and Δ*srtA* strains found associated with immortalized human oral keratinocytes (OKF6/TERT-2). A total of 2,921 cells derived from three separate experiments were visualized. (D) Relative hydrophobicity levels of S. gordonii Δ*ltaS* strain, compared to the WT, as determined by association with hexadecane. The bar represents the mean of results from six biological replicates ± SD.

Surface attachment and subsequent biofilm formation are vital for bacteria to persist in the oral environment (21–23) and require presentation of surface-associated proteins. Since the surface of the Δ*ltaS* mutant appeared irregular in comparison to that of the WT (Fig. 4), we investigated whether the ability of S. gordonii to attach to and/or form biofilms on saliva-coated surfaces was affected. Whereas the WT strain and Δ*ltaS* mutant showed similar initial attachment results (4.4 × 10^7^ ± 6.1 × 10^6^ CFU and 6.9 × 10^7^ ± 1.8 × 10^7^ CFU, respectively), the biofilms formed by the Δ*ltaS* strain were ∼67% less dense than those formed by the WT strain after overnight incubation (Fig. 4A). The reduction in biofilm biomass by the Δ*ltaS* mutant was similar to that previously seen with a Δ*srtA* strain, which fails to express many of the cell wall-anchored adhesive proteins (adhesins) (24) (Fig. 5A).

### (ii) Coaggregation.

S. gordonii may also contribute to the maturation of oral biofilms through direct cell-to-cell contacts with other members of the community, including Fusobacterium nucleatum, Porphyromonas gingivalis, and Actinomyces naeslundii (for a review, see reference 25). To determine whether LTA contributes to direct binding to other members of the oral community, we performed *in vitro* coaggregation assays. The loss of LTA was not reflected in any change in coaggregation phenotype or inhibition by l-arginine (Fig. 5B).

### (iii) Adherence to immortalized oral keratinocytes.

S. gordonii and other closely related oral streptococci attach to host cells (for a review, see reference 26). As modeled using group A streptococcus (GAS), LTA has been proposed to mediate initial interactions with human epithelial cells (27). Thus, we tested S. gordonii for LTA-dependent attachment to immortalized oral keratinocytes. The WT strain bound oral keratinocytes, whereas the Δ*ltaS* and Δ*srtA* strains did not (Fig. 5C).

### (iv) Surface hydrophobicity.

Although LTA has been reported to contribute to surface hydrophobicity in GAS (7), we observed no differences in surface hydrophobicity between the Δ*ltaS* and WT strains of S. gordonii (Fig. 5D).

### LTA deficiency affects expression and presentation of cell wall-associated proteins.

**FIG 6:**
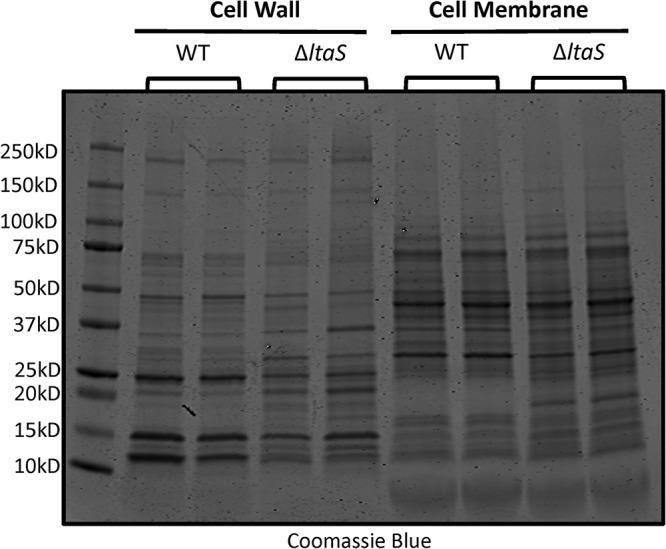
*ltaS* deletion affects cell wall-associated protein expression. Proteins (60 μg) from cell wall and cell membrane fractions from two biological replicates of WT and Δ*ltaS* strains were resolved on a 4%-to-20%-gradient SDS-PAGE gel and stained with GelCode Blue Safe protein stain.

LTA is an abundant component of the cell envelope of Gram-positive bacteria (4), and the *ltaS* deletion appeared to affect cell surface morphology (Fig. 4D to F) and select functions (Fig. 5). Therefore, we investigated the effect of *ltaS* deletion on the protein profiles of the membrane and cell wall. Cell membrane and cell wall fractions were collected and resolved on SDS-PAGE gels. The protein presentations in the WT and Δ*ltaS* cell membrane fractions were similar, whereas the protein profiles of the cell wall fractions were markedly different (Fig. 6).

**FIG 7:**
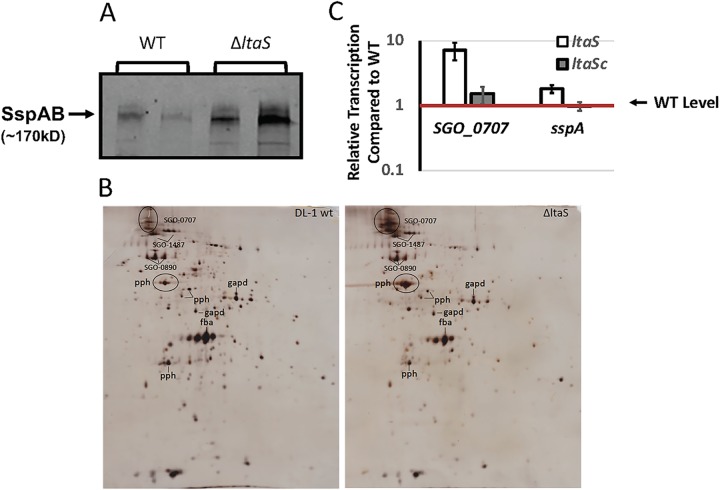
*ltaS* deletion affects SspAB and SGO_0707 expression. (A) Protein (60 μg) isolated from the cell wall fraction of two biological replicates of the WT and Δ*ltaS* strains was resolved on a 4%-to-20%-gradient SDS-PAGE gel and transferred to a nitrocellulose membrane. Levels of SspAB were detected by Western immunoblot analysis performed with the anti-P1 antibody. (B) Cell wall fractions (20 μg) of the WT and Δ*ltaS* strains were separated by isoelectric focusing (pH 4 to 7) in the first dimension and 7% SDS-PAGE in the second dimension. The identity of the major spots is shown. (C) Relative expression levels of SGO_0707 and *sspA* were determined during the exponential-growth phase in chemically defined medium (FMC) by qPCR analysis of total RNA extracted from the WT, Δ*ltaS*, and Δ*ltaS^c^* strains at 37°C. Bars represent means of results from six biological replicates ± SD.

Using label-free mass spectrometry (MS) analysis, we identified 80 proteins that were differentially expressed in the cell walls of the WT and the Δ*ltaS* strains. The analysis showed that 14 were more abundant in the Δ*ltaS* strain whereas 66 were more abundant in the WT (Table 1). Of particular interest, 5 LPXTG family proteins were more abundant in the cell wall fraction of the Δ*ltaS* strain (SGO_0430, SGO_0707, SGO_0890, SspA, and SspB). Using anti-P1 serum, which binds both SspA and SspB, we confirmed that the Δ*ltaS* strain showed greater SspAB abundance in the cell wall than the WT (Fig. 7A). Similarly, the Δ*ltaS* strain showed greater SGO_0707 abundance than the WT in the cell wall fraction as visualized using 2D gel electrophoresis (Fig. 7B). Therefore, in the WT strain, LTA appears to dampen SspAB and SGO_0707 presentation in the cell wall. To determine whether suppression occurs during or after transcription, we performed qRT-PCR analysis. The expression levels of *SGO_0707* and *sspA* in the Δ*ltaS* strain were 7-fold and 2-fold greater than those seen with the WT, respectively (Fig. 7C). Hence, expression of *SGO_0707* and *sspA* appears to be transcriptionally suppressed in the WT. Complementation of the Δ*ltaS* strain (*ltaS^c^*) restored transcription of *SGO_0707* and *sspA* to WT levels.

**TABLE 1:** List of differentially expressed proteins identified by mass spectrometry

Name	Locus	Annotation
Proteins more abundant in *ΔltaS* strain		
	SGO_1247	5’-Nucleotidase family protein
AckA	SGO_1916	Acetate kinase
	SGO_1069	Aminopeptidase
	SGO_0843	Carboxypeptidase
	SGO_0911	Hypothetical protein
	SGO_0430	LPXTG cell wall surface protein
	SGO_0707	LPXTG cell wall surface protein
	SGO_0890	LPXTG cell wall surface protein, collagen-binding domain
	SGO_1176	Peptide methionine sulfoxide reductase
PgK	SGO_0209	Phosphoglycerate kinase
DegP	SGO_2150	Serine protease
SspA	SGO_0210	Streptococcal surface protein A
SspB	SGO_0211	Streptococcal surface protein B
	SGO_1177	Thioredoxin family protein

Proteins more abundant in wild type		
	SGO_0953	2-Iminobutanoate/2-iminopropanoate deaminase
ThiJ	SGO_1434	4-Methyl-5(beta-hydroxyethyl)-thiazole monophosphate synthesis protein
	SGO_1860	5'-Nucleotidase, lipoprotein e(P4) family
	SGO_1342	ABC transporter, ATP-binding protein
ButA	SGO_1096	Acetoin dehydrogenase
	SGO_1862	Alkaline shock protein
	SGO_0578	Amino acid ABC transporter, permease protein
	SGO_0982	Amino acid ABC transporter, amino acid-binding protein
	SGO_0104	Arabinogalactan oligomer/maltooligosaccharide transport system substrate-binding protein
ArcA	SGO_1593	Arginine deiminase
	SGO_1082	Basic membrane protein A
	SGO_1630	Branched-chain amino acid transport system substrate-binding protein
	SGO_1626	Branched-chain amino acid transport system substrate-binding protein
FtsE	SGO_1440	Cell division transport system ATP-binding protein
	SGO_0823	Cof family protein
	SGO_0785	Cof family protein
	SGO_0059	Conserved hypothetical protein
	SGO_0957	Conserved hypothetical protein
AtpF	SGO_1546	F-type H^+^-transporting ATPase subunit B
PrsA	SGO_1572	Foldase protein
GlcK	SGO_1144	Glucokinase
GdhA	SGO_0276	Glutamate dehydrogenase
	SGO_1036	Glutamine transport system ATP-binding protein
	SGO_1037	Glutamine transport system substrate-binding protein
Gap	SGO_0207	Glyceraldehyde 3-phosphate dehydrogenase
	SGO_0390	Glycerol-3-phosphate dehydrogenase
	SGO_0164	Glycerol-3-phosphate dehydrogenase
	SGO_0832	Hypothetical protein
	SGO_1065	Hypothetical protein
	SGO_1677	Hypothetical protein
	SGO_0378	Hypothetical protein
	SGO_1232	l-Lactate dehydrogenase
	SGO_0652	Lon-like protease
	SGO_0372	Malolactic enzyme
RgfB	SGO_0506	Maltose 6'-phosphate phosphatase
	SGO_1283	Oxidoreductase
Pbp1a	SGO_0586	Penicillin-binding protein 1A
Pbp2a	SGO_2010	Penicillin-binding protein 2A
PgdA	SGO_0948	Peptidoglycan-N-acetylglucosamine deacetylase
ManB	SGO_1215	Phosphoglucomutase
	SGO_1149	Pneumococcal vaccine antigen A-like protein
	SGO_0457	Polar amino acid transport system substrate-binding protein
Wzd	SGO_2016	Polysaccharide export protein
Bta	SGO_1216	Possible bacteriocin transport accessory protein
	SGO_0599	PPM family protein phosphatase
PepQ	SGO_0771	Proline dipeptidase
	SGO_1580	PTS, cellobiose-specific IIB component^*a*^
PtcC	SGO_1576	PTS, cellobiose-specific IIC component
	SGO_1763	Putative aldouronate transport system substrate-binding protein
	SGO_1799	Putative endopeptidase
	SGO_0004	Putative lipoprotein
	SGO_0233	Putative lipoprotein
	SGO_0060	Putative membrane protein
	SGO_0140	Putative multi-antimicrobial-extrusion family transporter
ArcT	SGO_1589	Putative transaminase/peptidase
LytR	SGO_0535	Putative transcriptional regulator
Pyk	SGO_1339	Pyruvate kinase
	SGO_0667	Rhodanese family protein
	SGO_1338	Signal peptidase I
SrtB	SGO_2104	Sortase B
	SGO_1110	Surface antigen SCP-like domain
	SGO_0482	ThiJ/PfpI family protein
InfA	SGO_1963	Translation initiation factor IF-1
TpiA	SGO_0762	Triosephosphate isomerase
	SGO_1864	X-Pro aminopeptidase
	SGO_0521	YidC/OxaI family membrane protein insertase

a PTS, phosphotransferase system.

## DISCUSSION

LTA has long been viewed as an important component of the Gram-positive cell envelope, since inhibition of LTA synthesis leads to the death or impaired growth of many species (28–32). Why LTA is physiologically important to the cell, however, remains obscure. To start addressing this issue, we took advantage of a viable LTA-deficient strain of the human oral commensal S. gordonii. Our data implicate LTA as an important contributor to surface protein biogenesis, affecting the abundance of as many as 80 proteins under the conditions tested (Table 1). This widespread effect in surface protein presentation could easily explain the pleiotropic phenotypes associated with LTA loss.

### LTA synthesis and structure.

Differing from the complex LTA structure of Streptococcus pneumoniae, which displays a type IV LTA, the structure of the unusual type I LTA produced by S. gordonii is decorated with d-alanine and glucose, based on comparative 1D ^1^H NMR, ESI-MS, and carbohydrate compositional analyses (Fig. 2). Whereas LtaS is essential for many species, Bacillus subtilis and Bacillus anthracis encode four LtaS homologs which seem to perform overlapping and or redundant activities (reviewed in reference 6). S. gordonii differs from those species in that there is only one *ltaS* ortholog (*SGO_1377*) identified in its genome, and its deletion leads to complete loss of LTA as determined by Western immunoblotting (Fig. 1). Less obvious to us, however, are the components involved in the synthesis and translocation of the glycolipid anchor from the inner to the outer leaflet of the S. gordonii membrane. In some bacteria, or in mutant strains with deletions in the glycolipid synthesizing enzymes, LTA can be polymerized directly onto diacylglycerol (DAG) groups (33–35). In other bacteria, such as S. aureus, B. subtilis, S. agalactiae, and Enterococcus faecalis, LTA is polymerized on a Glc_2_-DAG glycolipid anchor (reviewed in reference 36). We searched the S. gordonii genome for homologs of the glucosyltransferases involved in Glc_2_-DAG synthesis in S. aureus, B. subtilis, S. agalactiae, and E. faecalis. We identified two proximal glucosyltransferase genes (*SGO_0774* and *SGO_0775*) with sequence similarities to GBS0683 and *ladA* of S. agalactiae. We are currently assessing the roles of SGO_0774 and SGO_0775 in Glc_2_-DAG and LTA synthesis and function.

Whereas Glc_2_-DAG synthesis occurs inside the cell, the addition of glycerol phosphate subunits onto the glycolipid anchor (and therefore LTA synthesis) occurs outside (37). In S. aureus, the membrane permease LtaA is required for the translocation of glycolipids across the membrane (30). In S. gordonii and other species, no enzyme with comparable function has been identified.

### A role in peptidoglycan maintenance and envelope stress response (ESR).

In many Gram-positive bacteria, LTA depletion is associated with cell growth defects (reviewed in reference 6). In S. aureus, for example, depletion of LtaS results in aberrant positioning of division septa, pointing to a link between LTA and cell division (32). Septum formation and cytokinesis are crucial steps in bacterial growth and are often regulated by FtsE and FtsX (38–41). In S. gordonii, loss of LTA decreases FtsE abundance, potentially affecting septum formation and contributing to the growth defect in our LTA-deficient strain (Fig. 3A). Interestingly, S. aureus, B. subtilis and Listeria monocytogenes cells are elongated when LTA synthesis is disrupted (reviewed in reference 6), whereas S. gordonii appears to show the opposite phenotype (Fig. 3B).

Peptidoglycan biosynthesis and remodeling are also important steps in cell division and in the overall maintenance of the cell wall (reviewed in reference 42). Cell wall maintenance requires the activity of multiple enzymes, including members of the widely conserved penicillin-binding protein (PBP) family. In S. aureus, LtaS interacts with Pbp1 and Pbp2 among other peptidoglycan biosynthesis and remodeling proteins (43). Similar interactions may also occur in S. gordonii, since Pbp1a and Pbp2a are less abundant in cell wall fractions derived from our LTA-deficient strain (Table 1). In S. aureus, depletion of Pbp1 induces incomplete septation and alterations in cell morphology but does not appear to alter peptidoglycan cross-linking (44, 45), whereas depletion of Pbp2 results in the presence of peptidoglycan that is significantly less extensively cross-linked (46, 47) and therefore weaker. Thus, the cell-rounding phenotype displayed by the Δ*ltaS* strain (see Fig. S2A and B in the supplemental material) and its increased sensitivity to osmotic pressure (Fig. S3A and B) might result from the decrease in the level of Pbp1a and/or Pbp2a in the cell wall.

The cell wall and the cell membrane (together referred to as the cell envelope) defend the cell against environmental insults. Thus, it is not surprising that most bacterial species employ multiple systems to monitor cellular integrity (reviewed in reference 48). As a major component of the Gram-positive cell wall and therefore the cell envelope, loss of LTA could impact cell envelope integrity and trigger an envelope stress response (ESR). In fact, LTA has been previously proposed to help stabilize the Gram-positive cell membrane (49).

In Gram-negative bacteria, the DegP serine protease is an important component of the ESR, degrading misfolded or aggregated cell envelope proteins (reviewed in reference 50). We observed that DegP abundance was greater in cell wall fractions from the *ltaS* mutant than in those from the WT (Table 1), perhaps signaling that the number of misfolded proteins had increased. The mechanism behind the increase in DegP abundance in the *ltaS* mutant remains unexplored. In Escherichia coli, *degP* transcription is regulated by the stress response two-component system (TCS) CpxAR (51). Despite significant progress in the past decade, ESR studies in Gram-positive bacteria still lag those in Gram-negative species. At this point, it is unclear if and how Gram-positive bacteria can sense the accumulation of misfolded proteins in their cell envelope. Accumulation of misfolded proteins could affect membrane composition triggering the activation of a TCS. In B. subtilis, for example, at least three of the four TCSs involved in ESR have sensor kinases with very short (<10 amino acids) extracellular loops connecting their two transmembrane domains (52). This domain architecture has been identified in at least one TCS in S. gordonii, SGO_1180 (53). Consistent with the hypothesis that SGO_1180 might be involved in mitigating an ESR induced by the loss of LTA, a Δ*ltaS* Δ*SGO_1180* double mutant seems to be lethal (B. P. Lima, unpublished data), suggesting that the ability of S. gordonii to cope with the loss of LTA requires SGO_1180.

### LTA and biofilm formation.

LTA contributes to biofilm formation in many species (8, 54–56). How loss of LTA affects biofilm formation, however, seems to differ from species to species. In S. aureus, for example, LTA is required for attachment to polystyrene surfaces, affecting the overall biofilm formation (8). In Enterococcus faecalis, however, loss of LTA does not affect surface attachment but severely affects biomass accumulation after the initial attachment (54). Similarly, our data show that loss of LTA in S. gordonii leads to a significant defect in biofilm formation (Fig. 4 and 5A) without any detectable impact in the initial attachment to saliva-coated polystyrene plates, suggesting a defect in a biofilm maturation step.

### Concluding remarks.

Although an LTA-deficient strain of S. gordonii has been constructed previously (13, 14, 57), the immune response to LTA was the primary focus of those studies. Here, we show that S. gordonii produces a complex type I LTA that shows abundant d-alanylation and glycosylation. This LTA plays an important role in cell surface protein biogenesis, key to cell envelope homeostasis.

## MATERIALS AND METHODS

### Bacterial strains and media.

All bacterial strains and plasmids used in this study are listed in Table 2. S. gordonii DL1 was grown in brain heart infusion (BHI) broth or on agar plates (Difco, Sparks, MD) or in chemically defined media (FMC) (58) at 37°C in 5% CO_2_ as indicated. Actinomyces naeslundii ATCC 12104 was grown in BHI broth or agar plates (Difco, Sparks, MD) at 37°C in 5% CO_2_. Fusobacterium nucleatum strain ATCC 23726 was grown anaerobically at 37°C in Columbia broth or on Columbia agar plates (BD Difco, Detroit, MI) supplemented with 5% defibrinated sheep blood (HemoStat Laboratories, Dixon, CA). Porphyromonas gingivalis A7436 was grown anaerobically on Columbia broth supplemented with 5% defibrinated sheep blood, 1 μg/ml hemin, and 1 μg/ml menadione. Escherichia coli was grown aerobically at 37°C in Luria-Bertani (LB) broth or on agar plates (BD Difco, Detroit, MI). When necessary, 50 μg/ml spectinomycin was added for selection of E. coli.

**TABLE 2:** Bacterial strains and plasmids used in this study

Strain or plasmid	Description (purpose)^*a*^	Source or reference
Strains		
*Escherichia coli* NEB 5-alpha	Competent *E. coli*—DH5α derivative	NEB
*Streptococcus gordonii* DL1	*S. gordonii* wild type	75
*Streptococcus gordonii* Δ*ltaS*::*JHMD1*	DL1 with in-frame deletion of *SGO_1377*	This study
*Streptococcus gordonii* Δ*ltaS*^c^	Complemented Δ*ltaS*::*JHMD1*; *SGO_1377* reinserted into the *attB* locus	This study
*Streptococcus gordonii* Δ*srtA*	DL1 with a markerless deletion of *srtA*	53
*Fusobacterium nucleatum* ATCC 23726	Wild type	ATCC
*Actinomyces naeslundii* ATCC 12104	Wild type	ATCC
*Porphyromonas gingivalis* 4612	Wild type	76

Plasmids		
pJHMD1	*S. gordonii ldh* promoter—ermAM-pheS*(A316G) in pUC57; Amp^r^ (markerless deletion system)	53
pFW5	*S. gordonii* shuttle vector, Spec^r^	77
pBPL11	pFW5 derivative containing *ltaS* ORF and its promoter (complementation)	This study

a Amp^r^, ampicillin resistance; ORF, open reading frame; Spec^r^, spectinomycin resistance.

### Construction of LTA-deficient mutant.

The Δ*ltaS* strain was constructed by allelic replacement of the nucleotide sequence that includes *ltaS* (*SGO_1377*) using the JHMD1 cassette from pJHMD1 as described previously (53). An ∼500-bp region immediately upstream and downstream of *ltaS* was amplified with primers *ltaS*-UP (Forw 5′-AAGAAAAGAGAGCATAGTCCC-3′ and Rev 5′-CTATGCTATGAGTGTTATCGTTTCTCGTTTTTTCACAAAAGTACTTCCTTG-3′) and *ltaS*-Down (Forw 5′-GTTATCTATTATTTAACGGGAGGAAATAAAGCAATACTTTGTCACACC-3′ and Rev 5′-TATACGAATTTATCCAAAAAAC-3′). Chromosomal DNA from S. gordonii was used as a template. The JHMD1 replacement sequence was amplified with primers JHMD1-Forw (5′-CGAGAAACGATAACACTCATAGCATAG-3′) and JHMD1-Rev (5′-TTATTTCCTCCCGTTAAATAATAGATAAC-3′) (53) and fused to the upstream and downstream flanking regions by splice overlap extension PCR (SOE-PCR) as described previously (59). The PCR product was then transformed into WT S. gordonii and plated on Todd-Hewitt (TH) agar plates containing 5 μg/ml erythromycin and incubated under anaerobic conditions (10% H_2_, 10% CO_2_, 80% N_2_) at 37°C as described previously (53).

### LTA purification and characterization.

S. gordonii was grown in FMC medium at 37°C in 5% CO_2_. LTA was purified from the WT biomass (8 liters of stationary-phase culture) and analyzed as described previously (60, 61). As judged by NMR, the purified LTA preparations did not contain high levels of lipoprotein or phospholipid, but the presence of trace levels of these contaminants cannot be ruled out. For de-d-alanylation, purified LTA (0.5 mg) was incubated in 5 mM ammonium bicarbonate (pH 8.5) for 24 h at room temperature, followed by freeze-drying to remove ammonium bicarbonate. LTA was then resuspended and dialyzed against MilliQ distilled water (dH_2_O) using cellulose ester dialysis membranes (Spectrum Labs) (molecular weight cutoff [MWCO], 0.5 to 1.0 kDa) to remove free d-alanines. LTA (1 mg) was monomerized by treatment at room temperature for 20 h with 100 μl of 47% hydrofluoric acid (HF), which was then evaporated under a filtered air stream and resuspended in 200 μl of ammonium bicarbonate. LTA monomers were further neutralized with dilute ammonium hydroxide and lyophilized.

LTA monomers were suspended in electrospray ionization mass spectrometry (ESI-MS) mobile phase (50% acetonitrile, 50% aqueous 10 mM ammonium acetate) and analyzed using a Waters 2695 high-performance liquid chromatography (LC) system and flow injection at a rate of 0.15 ml/min. Nitrogen drying gas was used at a setting of 300°C at 7 liters/min with capillary voltage of 2.8 kV.

Samples were subjected to acidic methanolysis and analyzed for glycosyl composition by combined gas chromatography/mass spectrometry (GC/MS) analyses of the per-*O*-trimethylsilyl (TMS) derivatives of the monosaccharide methyl glycosides as described previously (62).

### Whole-genome sequencing.

S. gordonii WT and Δ*ltaS* strains were grown in BHI broth at 37°C and 5% CO_2_ for 18 h. Genomic DNA was isolated using a Wizard genomic DNA isolation kit (Promega). To sequence paired-end reads, the University of Minnesota Genomics Center created 5 dually indexed Nextera XT libraries from 500 ng genomic DNA. The libraries were combined into a single pool and sequenced using a MiSeq PE Nano sequencer (v2; 250 bp), generating ≥1 million pass filter reads for the run. All expected barcodes were detected and well represented. The mean quality scores were ≥Q30 for all libraries. Reads were mapped against the S. gordonii genome (NC_009785_1) using Burrows-Wheeler Aligner version: 0.7.17-r1188. Using the CLC Genomics suite, binary alignment map (BAM) files were used to extract consensus sequences with a minimum coverage of 5 reads without the inclusion of the reference strain. Single nucleotide polymorphisms (SNPs) distinguishing the WT and Δ*ltaS* strains were manually identified using Integrative Genome Viewer.

### Δ*ltaS* mutant complementation.

The Δ*ltaS* strain was complemented by insertion of the endogenous *ltaS* gene with its native promoter into the *attB* site of the S. gordonii genome. The *ltaS* gene and its promoter were amplified from the WT S. gordonii strain using primer pair *latS-*For (5′-ATTCTAAATTATATCAAATATTGAGAAATATTT C3’) and *ltaS-*Rev (5′-TCATTGCTTACTAGAAGAAG-3’). The entire *attB* sequence was amplified using primer pair *attB*-Forw (5′-AAGGCATTTGTCTTTAATTCTACTG-3′) and *attB*-Rev (5′-AACCTGATTTATCAGGAAGC3-3’). The *attB* fragment was fused to *ltaS* and inserted by Gibson assembly (New England Biolabs, Ipswich, MA) into pDL278, which had been digested previously with enzymes BamHI and SalI (New England Biolabs, Ipswich, MA). The resulting plasmid was named pBPL11.

### Saliva collection and preparation for adhesion and biofilm assays.

Stimulated whole saliva was collected and pooled from at least three healthy, medication-free adult volunteers using protocols that were reviewed and approved by the Institutional Review Boards from the University of Minnesota and Malmo University. Saliva was processed as described previously (53) and sterilized by exposure to UV irradiation for 30 min (Spectroline UV Crosslinker FB-UVXL-1000; Spectronics, Westbury, NY). After a 48-h incubation at 37°C in 5% CO_2,_ sterilization was confirmed by plating saliva on BHI agar and enumerating CFU.

### Adhesion to saliva-coated surfaces.

The bottoms of 12-well plates (Corning Costar catalog no. 3513) were coated with 500 μl of sterilized saliva. To assess surface attachment, 1 ml of WT S. gordonii or Δ*ltaS* cells (adjusted to an optical density at 600 nm [OD_600_] of 1 in FMC broth) was added to each saliva-coated well. and the plates were incubated at 37°C for 45 min in 5% CO_2_ (∼1/2 S. gordonii doubling time under the conditions tested), avoiding rounds of cell division. After incubation, the FMC medium was aspirated and each well was washed twice with 1 ml of sterile phosphate-buffered saline (PBS), which was removed by aspiration. Adherent cells were scraped from the bottom of the wells using cell scrapers (Sarstedt, Inc., Newton, NC, USA) (25-mm overall length), resuspended in 1 ml of sterile PBS, serially diluted in 10-fold dilutions, and enumerated as CFU on BHI agar plates.

### Biofilm assay.

To assess biofilm formation, the S. gordonii WT and Δ*ltaS* strains were inoculated (200 μl of a 1:100 dilution of an overnight culture) into saliva-coated 96-well round-bottom plates and grown in FMC broth at 37°C for 16 to 18 h in 5% CO_2_. Biofilm formation was assessed by crystal violet retention as described previously (53).

### Coaggregation.

Interspecies coaggregation assays were performed in coaggregation buffer (CAB) containing 150 mM NaCl, 1 mM Tris, 0.1 mM CaCl_2_, and 0.1 mM MgCl_2_ as described previously (63, 64). Coaggregation was inhibited by adding 50 mM l-arginine to the suspensions of S. gordonii strains and vortex mixing after adding the partner strains.

### Adherence to immortalized oral keratinocytes.

Immortalized human oral keratinocytes (OKF6/telomerase reverse transcriptase 2 [TERT-2]) were grown as described previously (65) with modifications. Briefly, keratinocytes (1 × 10^5^ cells) were seeded on a sterile Fisherbrand microscope cover glass in wells of Costar 3524 24-well plates in 500 μl Gibco K-SFM and grown for 24 h at 37°C in 5% CO_2_.

The WT, Δ*srtA*, and Δ*ltaS* strains were grown overnight at 37°C in 5% CO_2_ and adjusted to a multiplicity of infection (MOI) of 100:1, with 10 μl of bacteria added to each well containing keratinocytes. Cultures were incubated for 30 min at 4°C to minimize internalization of bacteria, the medium was aspirated, wells were washed with 500 μl PBS and aspirated, and a LIVE/DEAD *Bac*Light bacterial viability kit (Life Technologies) was added to each well according to the manufacturer’s protocol. After a 15-min incubation at room temperature in the dark, the PBS containing the stain was aspirated from the wells. To visualize and count attached bacteria, mounting oil (Life Technologies) (2 μl) was placed onto a glass slide (Matsunami Glass Industries), and the glass slide was covered with a cover glass from the experimental wells (described above) and secured in place using clear nail polish.

### Hexadecane binding assay.

Hydrophobicity was assessed by hexadecane binding as described previously (66), with minor modifications. S. gordonii was grown in FMC medium overnight at 37°C in 5% CO_2_. An overnight culture was adjusted to an OD_600_ of 1.0, 1 ml was placed into a glass tube, and 100 μl of hexadecane (Sigma-Aldrich, St. Louis, MO) was added. The mixture was subjected to vigorous vortex mixing for 2 min and then allowed to stand for 10 min at room temperature to facilitate phase separation. The OD_600_ of the lower aqueous phase was recorded. Percent hydrophobicity was calculated using the following formula: percent hydrophobicity = [1 − (OD_600_ after vortex mixing/OD_600_ before vortex mixing)] × 100.

### Cell fractionation.

S. gordonii was fractionated as described previously (67) with minor modifications. Cells were grown in FMC medium and pelleted, and protoplasts were harvested as follows. Protoplasts and supernatants were separated by centrifugation at 12,000 × *g* for 20 min at 4°C. Supernatants were collected and dialyzed against 2 liters of 0.2% (wt/vol) Na_2_EDTA for 4 h followed by 2 liters of distilled water overnight and concentrated by centrifugation using an Amicon Ultra-15 Centrifugal Filter Untracel (Tullagreen, Carrigtwohill, Co. Cork, Ireland) (10K), and the product was designated the cell wall fraction. Protoplasts were resuspended in 10 ml of bacterial lysis buffer (BugBuster [EMD Millipore Corp., Billerica, MA]) and lysed by sonication (75 W for 8 min), and insoluble materials were removed by centrifugation (12,000 × *g*, 20 min, 4°C). Supernatants were collected, and membrane fractions were separated from cytoplasmic fractions using ultracentrifugation at 120,000 × *g* for 90 min at 4°C. Pellets and supernatants were collected separately. The supernatant was designated the cytoplasmic fraction. The pellet was resuspended in lysis buffer and designated the membrane fraction. Protein concentrations for each fraction were determined using Pierce bicinchoninic acid (BCA) protein assay (Thermo Scientific, Rockford, IL). To compare protein profiles, 60 μg of total protein from each fraction was resolved by 4% to 20% gradient SDS-PAGE and stained with GelCode Blue Safe protein stain (Thermo Scientific, Rockford, IL).

### Label-free quantification. (i) In-gel trypsin digestion.

A total of 60 μg of protein from each biological replicate was resolved by SDS-PAGE and digested as described previously (68).

**(ii) Liquid chromatography and mass spectrometry.** Triplicate WT and Δ*ltaS* samples were analyzed in random order. Approximately 1 μg of peptide mixture was injected for each sample. After trypsin digestion, the peptide mixtures were resolved using capillary liquid chromatography-tandem mass spectrometry (LC-MS/MS) on a Velos Orbitrap system (Thermo Scientific, Waltham, MA) (69) modified as follows. The capillary column dimensions were 100-μm internal diameter by 14-cm length; the flow rate for direct column load was 1.1 μl/min; the minimum signal intensity for precursor ion trigger was 15,000 counts (lock mass was not used); the dynamic exclusion (DE) duration was 30 s.

**(iii) Database searching and quantification.** We analyzed the tandem MS data in PEAKS Studio 8.5 (Bioinformatics Solutions, Inc., Waterloo, Ontario, CA) with the Quant module for protein detection and label-free quantification. The database search parameters were set as follows: no corrections for charge or precursor; no merge options and no spectrum filter applied; *de novo* precursor mass error tolerance of 50.0 ppm and fragment mass error tolerance of 0.1 Da; *de novo* enzyme trypsin and variable modifications for methionine oxidation and carbamidomethyl cysteine; maximum of 3 variable posttranslational modifications (PTMs) per peptide; PEAKS DB (database search) precursor mass error tolerance of 50.0 ppm and *de novo* fragment mass error tolerance of 0.1 Da; monoisotopic search type with trypsin enzyme, 1 missed cleavage site and amino acid modifications identical to *de novo* settings; S. gordonii (taxon ID 1302) protein database from NCBI reference sequence (downloaded 16 October 2017) merged with the common laboratory contaminant proteins from http://www.thegpm.org/crap/; PEAKS PTM parameters for deamidation (NQ), dioxidation (M), pyro-glutamic acid from Q, and N-terminal acetylation and oxidation (HW) with a maximum of 3 variable modifications per peptide; false-discovery-rate estimation enabled. For quantification, we set the following parameters: PEAKSQ significance method; protein signification value of ≥20; minimum fold change = 2; total ion chromatogram for the normalization method. For comparisons, the six samples (triplicates for each condition) were assembled into 2 groups. Details of the retention alignment algorithm (70) and PEAKS Q significance scores were as described previously (71).

### Two-dimensional polyacrylamide gel electrophoresis.

Two-dimensional (2D) polyacrylamide gel electrophoresis resolved a volume corresponding to 20 μg of cell wall protein as described previously (72). The protein concentration was determined using a 2D Quant kit (GE Healthcare Life Sciences). All gels were run in triplicate. Only protein spots detected in all replicates were identified.

### Identification of proteins on 2D gels using LC-MS/MS.

Proteins of interest were excised manually from Coomassie brilliant blue-stained gels and trypsin digested, and peptides were separated using LC and characterized using MS/MS as described previously (72). Mass lists were used as the input for Mascot MS/MS ion searches of the NCBInr database using the Matrix Science Web server.

### Western immunoblot analysis.

S. gordonii cultures were harvested and fractionated as described above, and 10 μg of protein from each fraction was analyzed using Western immunoblotting. To detect S. gordonii LTA, mouse monoclonal antibody against Gram-positive bacterial LTA (G43J; Thermo Fisher Scientific, Rockford, IL) was used at a 1:50 (vol/vol) dilution. To detect S. gordonii SspA and SspB surface proteins, rabbit anti-S. mutans P1 serum diluted 1:1,000 (vol/vol) was incubated overnight at 4°C as described previously (24). Blots were incubated for 1 h at room temperature with IRDye 680RD goat anti-mouse and IRDye 800CW goat anti-rabbit secondary antibody (Li-Cor, Lincoln, NE), respectively, and immunoreactive proteins were visualized. Total protein transferred onto the nitrocellulose membrane was quantified using REVERT total protein stain (Li-Cor, Lincoln, NE) according to the manufacturer’s instructions.

### Total RNA purification and RT-qPCR.

Overnight cultures were diluted 1:100 (vol/vol) in 5 ml of sterile FMC media in 15-ml polypropylene conical vials (Sarstedt) and incubated overnight at 37°C in 5% CO_2_. Cultures were pelleted at 4,300 × *g* at 4°C, and supernatants were removed. Total RNA purification, cDNA synthesis, and multiplex real-time quantitative PCR (RT-qPCR) were performed as described previously (53).

### Scanning electron microscopy.

Biofilms were allowed to form on saliva-coated hydroxyapatite disks (Clarkson Chromatography Products Inc., South Williamsport, PA) (9.65 mm by 1.52 mm) for 12 h, dehydrated, and processed as described previously (73). The samples were viewed with a field-emission-gun scanning electron microscope (FE-SEM) (6500; JEOL, Tokyo, Japan) (74).
