# Supplementary figures and images for: Streptococcus gordonii Type I Lipoteichoic Acid Contributes to Surface Protein Biogenesis
Source: mSphere. 2019 Dec 4;4(6):e00814-19. doi: 10.1128/mSphere.00814-19 (PMC6893214; doi:10.1128/mSphere.00814-19)

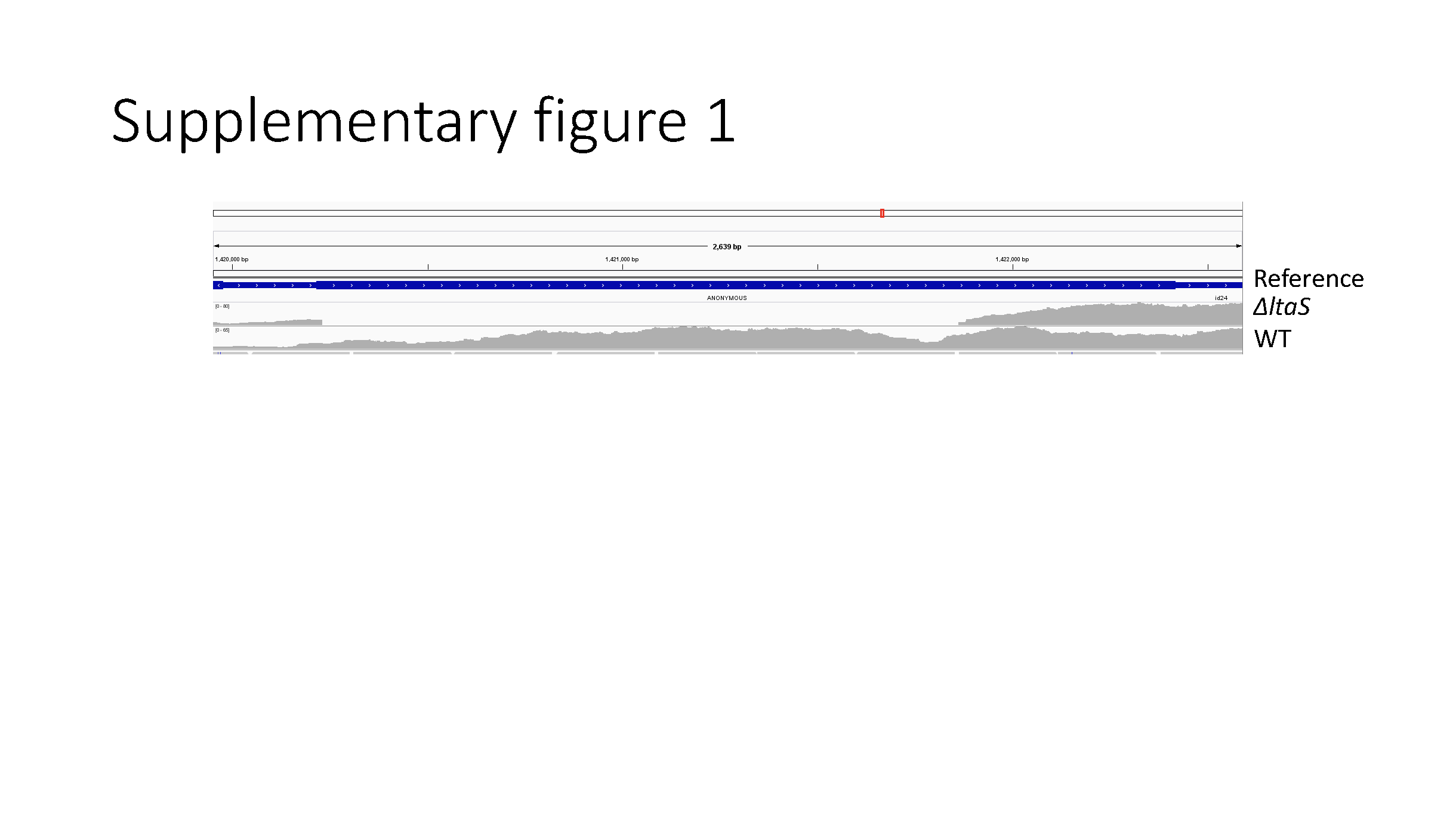

Supplement: FIG S1 [file mSphere.00814-19-sf001.tif]

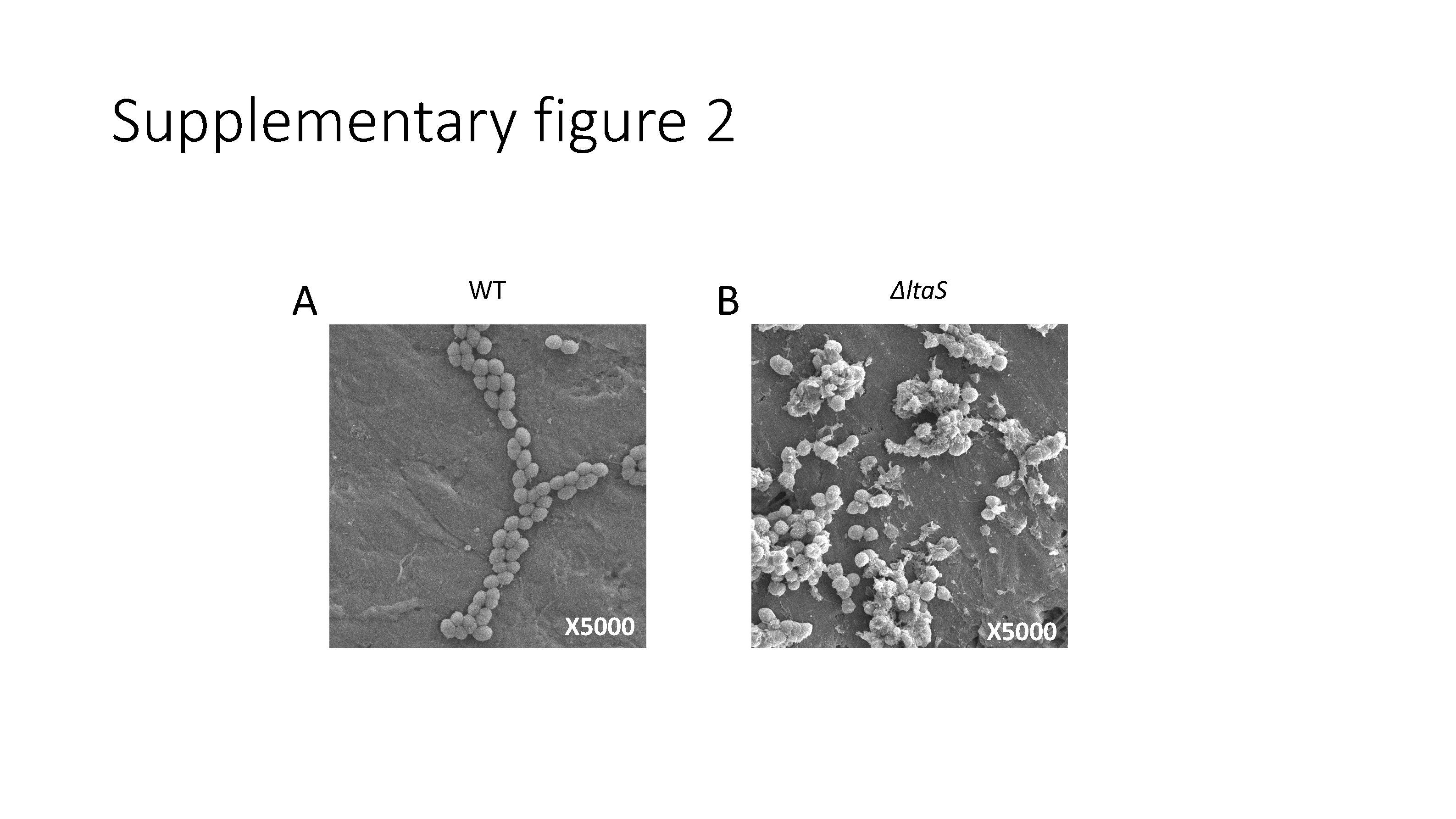

Supplement: FIG S2 [file mSphere.00814-19-sf002.tif]

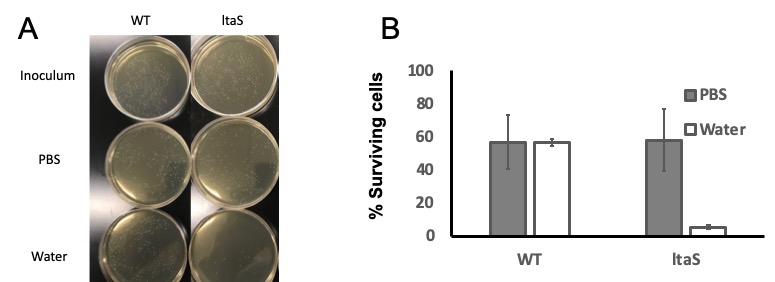

Supplement: FIG S3 [file mSphere.00814-19-sf003.jpg]
